# Enlarged Reactional Periostitis of the Peroneal Tubercle Mimicking Osteochondromatosis of the Calcaneus: A Case Report

**DOI:** 10.7759/cureus.25429

**Published:** 2022-05-28

**Authors:** Michail Vavourakis, Meletis Rozis, Athanasios Galanis, Dimitrios Zachariou, Ioannis Kolovos, Christos Patilas, Christos Eftychiadis, Spyros G Pneumaticos

**Affiliations:** 1 3rd Orthopaedic Department, National and Kapodistrian University of Athens, KAT General Hospital of Athens, Athens, GRC; 2 Department of Pathology, KAT General Hospital of Athens, Athens, GRC

**Keywords:** foot osteochondromatosis, foot and ankle tumors, peroneal tendon injury, calcaneal tubercle hypertrophy, reactional periostitis

## Abstract

Foot and ankle tumors are relatively rare. Nevertheless, the calcaneus is a prevalent location accommodating various lesions. Reactional periostitis of the lateral wall is rarely encountered but can potentially mimic a wide variety of tumors. We present a case of excessive proliferation due to chronic compression of the peroneal tendons against the calcaneus in a female patient with a history of diminished foot control, treated successfully by tumor excision and peroneal restoration via the tubularization technique. This study aimed to underline the mimicking potential of reactional periostitis and its effect on the peroneal tendons and hindfoot motion.

## Introduction

Calcaneal tumors and tumorous entities are relatively rare, comprising approximately 3% of foot and ankle osseous lesions [1]. Therefore, a delay in diagnosis and treatment planning is typical, as most clinicians are unfamiliar with those lesions [2-4]. Differential diagnosis includes benign and malignant tumors, reactive conditions, and tumor-like lesions, depending on the radiographic appearance and patient's medical history. Intermittent pain exacerbating during the night, swelling, and palpation of a well-defined mass are some clinical manifestations of these lesions. Although computed tomography (CT) and magnetic resonance imaging (MRI) help define cortical and mineralization changes, definitive diagnosis is typically obtained via tissue biopsy.

Reactional periostitis, an inflammatory entity of the periosteum provoking woven bone deposition, may affect the calcaneal peroneal tubercle (CPT). This may be a result of various conditions such as trauma, arthritic changes, infection, tumors, and certain drugs (e.g. teriparatide). The appearance of a periosteal reaction is determined by the intensity, aggressiveness, and duration of the underlying insult [5]. A wide range of causative factors and, occasionally, a confusing radiological appearance could mislead the diagnosis.

We present a case of excessive CPT osseous proliferation due to chronic compression of the peroneal tendons against the calcaneus in a female patient with a history of diminished foot control following spinal surgery. The purpose of this article is to underline the mimicking potential of reactional periostitis and its effect on the peroneal tendons and hindfoot motion.

## Case presentation

A 65-year-old female patient was admitted due to a painful mass on the lateral aspect of her right hindfoot. Regarding her medical history, she reported a right drop foot following a spinal surgery 10 years ago, which eventually improved over time. Seven years ago, she noticed a lump on the lateral side of her foot, around the calcaneus, that was asymptomatic. Nevertheless, the same mass was reported enlarging through time, causing gait difficulties and becoming painful during ambulation.

**Figure 1:**
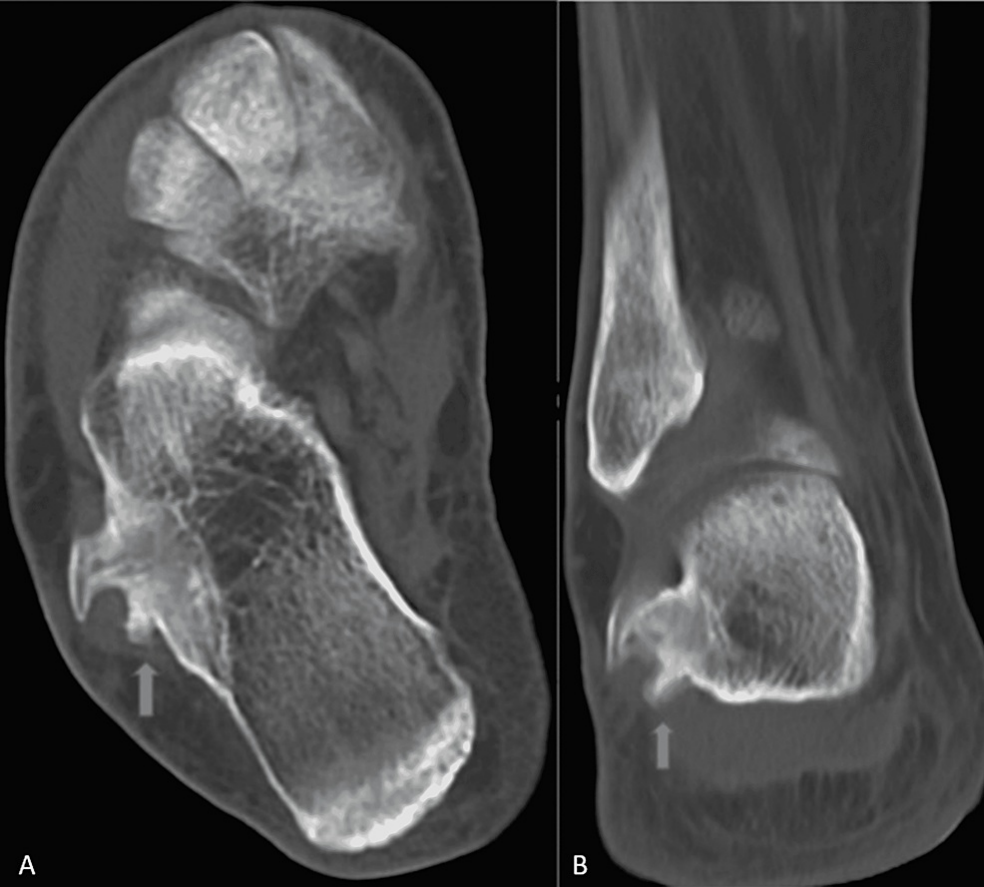
Bony overgrowth of calcaneal peroneal tubercle on CT scan. A: axial plane; B: coronal plane.

Clinical examination indicated reduced eversion strength (3+ out of 5), but no further signs suggesting a drop foot lesion. Meanwhile, the pain was elicited upon direct palpation of the peroneal tendons. Plain radiographs and CT images showed a bony overgrowth originating from the CPT, separating the peroneal tendons and pushing them off the calcaneus (Figure 1).

**Figure 2:**
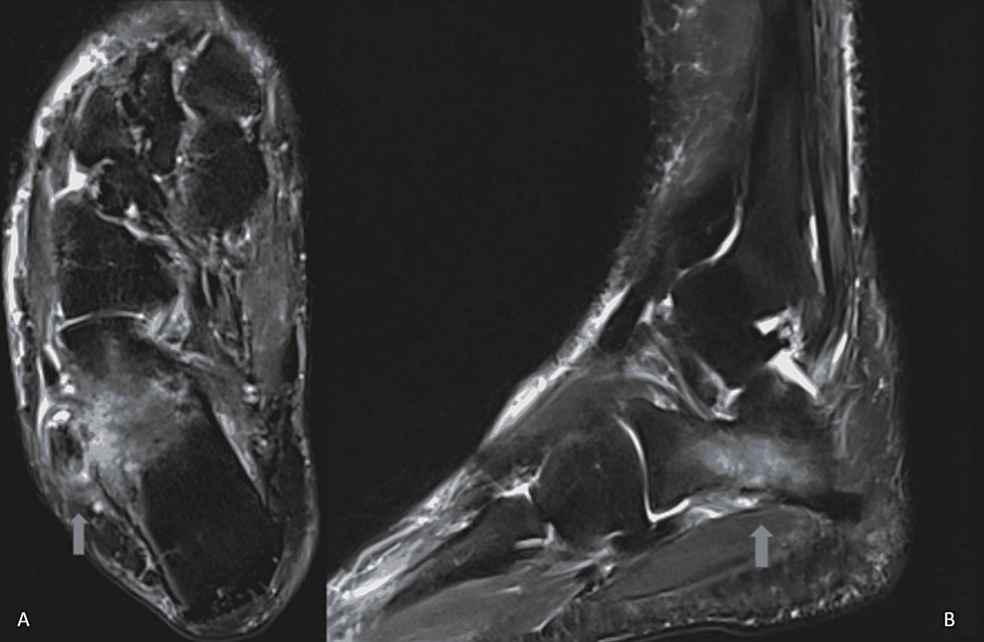
Preoperative MRI depicting an osseous lesion accompanied by diffuse bone marrow edema. A: axial plane; B: sagittal plane.

A subsequent MRI revealed diffuse bone marrow edema accompanied by a low-intensity lesion on T2-weighted images (Figure 2). No apparent cartilaginous overlay was observed. In addition, extended longitudinal ruptures were present on both the peroneal tendons.

Due to the long duration of symptoms and radiological features, it was decided to proceed with surgical treatment for pain relief. A curved lateral incision parallel to the peroneus longus was performed. The sural nerve was exposed and protected proximally. After that, we incised the superior peroneal retinaculum (SPR), identifying the peroneal tendons and dislocating them off the peroneal tubercle. An osteotome was used to remove the proliferative tubercle at the same level of the calcaneal lateral wall. Both peroneal tendons were debrided of scar tissue and sutured using the tubularization technique with absorbable sutures [6]. The excised lesion involving the floor of both peroneal sheaths was sent for biopsy. Finally, the SPR was sutured back in place and its tension was tested to avoid retromalleolar dislocation throughout the ankle's range of motion, while the surgical wound was closed as usual.

**Figure 3:**
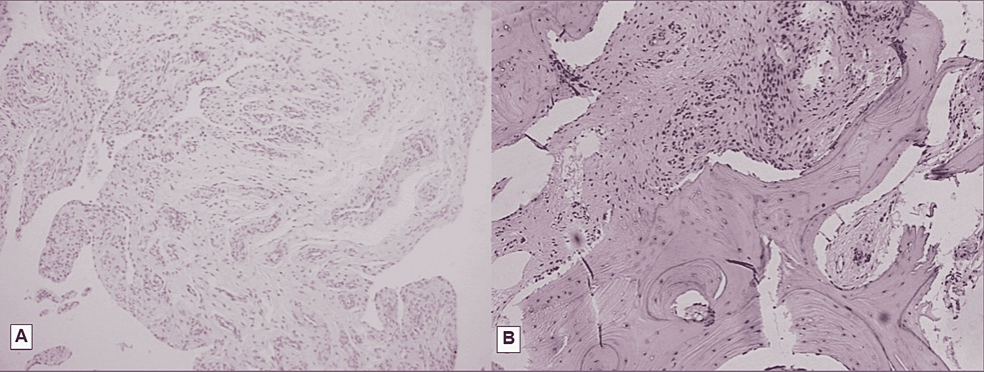
A: Synovial membrane demonstrating mild signs of hyperplasia and chronic inflammation, and presence of vascular hyperplasia. B: Spindle-shaped fibroblasts, adjacent to masses of woven bone, with the presence of cement-line lines and osteoblastic activity (hematoxylin and eosin, magnification × 100).

Postoperatively, a weight-bearing cast was applied for three weeks. There were no complications regarding wound healing. Sutures were removed three weeks later and the patient was allowed to fully weight-bear without restrictions after the fourth week. Histological evaluation of the lesion revealed chronic inflammatory synovial proliferation and bone remodeling, typical findings of reactional periostitis (Figure 3).

**Figure 4:**
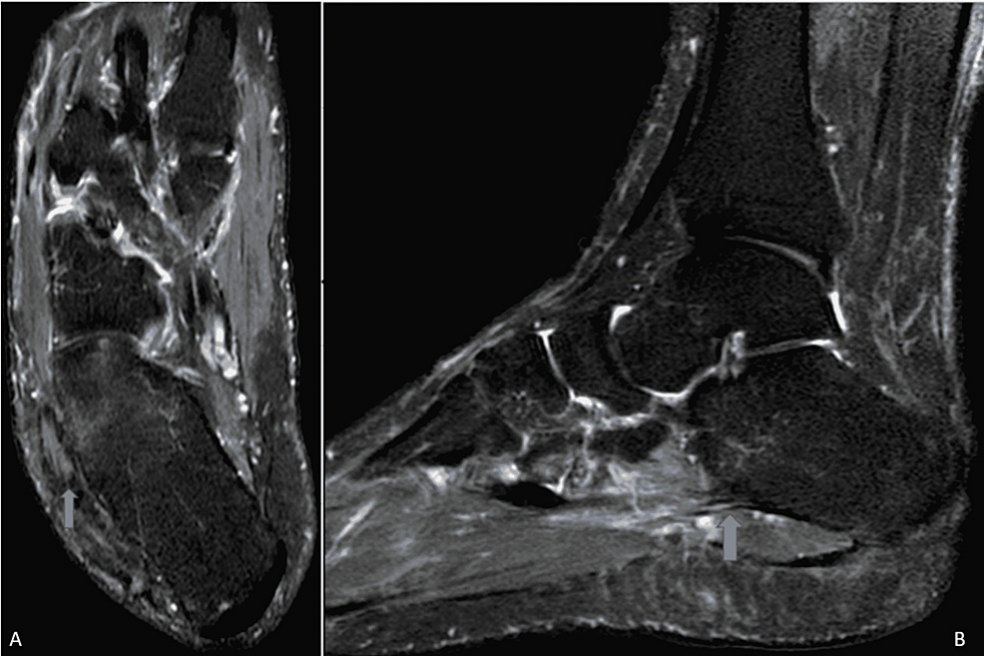
MRI images three months postoperatively revealed complete excision of the osseous lesion and a significant decrease in bone marrow edema. A: axial plane; B: sagittal plane.

At the six-month follow-up visit, eversion strength had significantly increased, with the patient reporting substantial pain improvement, even after prolonged walking. More specifically, the preoperative American Orthopaedic Foot And Ankle Society (AOFAS) score of 71 points (out of 100 maximum) significantly improved to 95 points, while a preoperative visual analog scale (VAS) pain score of 8 points (out of 10 maximum) improved to 2 points. Eversion strength was estimated to be 4+ from 3+ (out of 5) before her operation. In addition, a follow-up MRI (Figure 4), on the third month postoperatively, revealed only mild marrow edema with indications of ongoing peroneal tendon healing.

## Discussion

Tumors of the foot and ankle demonstrate a vast mimicking potential, so surgeons should always be aware of the local anatomy to identify lesions with similar clinical and radiological features. Osteoid osteoma, a benign self-limiting tumor with a diameter less than 1.5 cm, has a typical radiographic appearance with a well-defined intracortical lucency and surrounding sclerosis and is usually accompanied by night pain, relieved by nonsteroidal anti-inflammatory drugs (NSAIDs) [7-9]. Osteoblastoma, an uncommon benign bone-forming neoplasm that accounts for about 1% of all primary bone tumors, is usually larger than 1.5 cm, potentially behaving aggressively by expanding to nearby tissue, and may appear as a radiographically destructive moth-eaten or permeative lesion. The pain associated with an osteoblastoma is typically not relieved by NSAIDs [10,11]. Our patient had not complained of night pain. In addition, although both lesions can cause an extensive periosteal reaction and bone marrow edema, they have a characteristic nidus and do not cause further osseous proliferation of the calcaneus lateral wall structures.

On the other hand, osteochondromas (cartilage-capped bony projections or outgrowth on bones' surface) share similar radiological features with the peroneal tubercle overgrowth, including a continuous cortex with a medullary cavity that flows into the lesion. In addition, there may be a stalk (pedunculated) or a broad connection to the cortex (sessile) along with cartilaginous coverage. In our case, the lesion was located on the lateral calcaneal wall with diffuse bone marrow edema. Nevertheless, neither a demarcated cavity nor chondral overlay was identified on the MRI.

We did not obtain a preoperative biopsy on this patient. The reason is that, as concluded by the above differential diagnosis, both her medical history and the radiological features were indicative of a benign tumor. Therefore, although excluding the diagnosis of an osteochondroma was not definite preoperatively, the treatment remains the same as in reactive periostitis [12,13]. In addition, the MRI's characteristics of cartilaginous absence on the lesions' borders minimized the possibility of malignant transformation that can rarely occur with osteochondromas [14,15].

The peroneal tubercle separates the peroneus longus and brevis tendons. Hypertrophy may occur in response to trauma, physical overactivity, or inflammatory changes related to peroneal tendon activity, resulting in peroneal misalignment. In patients with pes cavus, this chronic pressure of peroneal tendons against the lateral calcaneal wall may result in reactive periostitis and thus hypertrophy of the peroneal tubercle [16-18]. In our case, the leading cause was assumed to be poor foot and ankle control, established after her spinal surgery. Although we restored the damaged peroneal tendons and improved the lever arm by removing the tumor, gait abnormalities were not fully restored as they were attributed to her older spinal lesion. Moreover, a hypertrophic peroneal tubercle may affect the peroneal tendons causing a stenosing tenosynovitis or even a partial/full-thickness tear, thus diminishing their function and inflicting pain [17-19]. In this case, longitudinal tears were present on both tendons, so the tubularization technique's direct repair was the only available fixation option [20].

## Conclusions

In conclusion, despite being a rare entity, it should be noted that the distinction of foot and ankle tumors is crucial as they demonstrate a vast mimicking potential. Suspicion of a possible malignant lesion should always be raised, while knowledge of the local anatomy and the clinical and radiological features of each specific tumor is essential to apply the appropriate treatment without delay. Reactional periostitis, usually produced by chronic peroneal pressure against the lateral calcaneal wall, although a benign condition, can be a severely limiting factor in patients' everyday routine, causing constant pain and gait abnormalities. It is vital to be adequately diagnosed and treated early, as removing the tumor and restoring the damaged peroneal tendons can improve life quality by inducing pain relief and improving the patient's lever arm.
